# Oral food challenges in FPIES: A narrative review and proposals for emergency and home‐based management of acute FPIES reaction

**DOI:** 10.1111/pai.70402

**Published:** 2026-07-19

**Authors:** Virginie Jubin, Pascale Dumond, Grégoire Benoist, Flore Amat, Harriet Corvol, Laure Couderc‐Kohen, Antoine Deschildre, Angélique Doc, Amandine Divaret‐Chauveau, Kamal El Abd, Agnès Linglart, Carine Metz‐Favre, Anne Hoppe, Karine Levieux, Martine Morisset, Frederic Valla, Sibylle Blanc, Anaïs Lemoine, the French Working Group Comprising the SFA (French Allergy Society), and the GFRUP (French‐speaking Pediatric Resuscitation and Emergency Group) and the Sp2A (French Society of Pediatric Pneumology and Allergology) of the SFP (French Pediatric Society)

**Affiliations:** ^1^ Société Française d’Allergologie (SFA) French FPIES working group Montpellier France; ^2^ Cabinet médical Medilac Le Bourget du Lac France; ^3^ Hôpital Couple Enfant La Tronche France; ^4^ Pôle Médical Saint Martin Blénod‐lès‐Pont‐à‐Mousson France; ^5^ Pediatric Allergy Department Hôpital d'enfants, CHRU de Nancy Vandoeuvre les Nancy France; ^6^ Service de Pédiatrie Générale, HDJ Allergologie pédiatrique Hôpital Ambroise Paré, AP‐HP Boulogne‐Billancourt France; ^7^ Société Française d’Allergologie (SFA) Food Allergy working group Montpellier France; ^8^ Pediatric Pulmonology and Allergology Department, Robert Debré Hospital, AP‐HP Université Paris Cité Paris France; ^9^ INSERM 1018, Respiratory Integrative Epidemiology Villejuif France; ^10^ Société de Pneumologie et d'Allergologie Pédiatrique (SP2A) Paris France; ^11^ Pediatric Pneumology Hôpital Trousseau, AP‐HP Paris France; ^12^ Sorbonne University, Inserm U938, Saint‐Antoine Research Center (CRSA) Paris France; ^13^ Pédiatrie médicale et médecine de l’adolescent CHU de Rouen Rouen France; ^14^ Pediatric Pulmonology and Allergy Unit, CHU Lille ‐ Jeanne de Flandre Hospital University of Nord de France Lille France; ^15^ Univ. Lille, CNRS, Inserm, CHU Lille ‐ Jeanne de Flandre Hospital, Institut Pasteur de Lille, U1019‐UMR 9017‐CIIL‐Center for Infection and Immunity of Lille Lille France; ^16^ Société Française d’Allergologie (SFA) AllergoDiet working group France; ^17^ Dietetician unit, CHRU de Besançon Besançon France; ^18^ UR 3450 DevAH, Health Faculty Nancy France; ^19^ Pediatric Allergology Center, Department of Pediatrics CHC MontLégia Clinic Liège Belgium; ^20^ Société Française de Pédiatrie (SFP) Paris France; ^21^ Endocrinology and Diabetes for Children, Physiologie et Physiopathologie Endocriniennes, Bicêtre Paris Saclay Hospital, Le Kremlin Bicêtre Université Paris‐Saclay, Inserm, AP‐HP France; ^22^ Allergy Division, Department of Chest Diseases University Hospital Strasbourg France; ^23^ Pediatric Pulmonology and Allergology Department, CHU d’Angers Angers France; ^24^ Groupe Francophone de Réanimation et Urgences Pédiatriques (GFRUP) Paris France; ^25^ Services d'urgences pédiatriques CHU de Nantes Nantes France; ^26^ Nutrition support team Hospices Civils de Lyon Lyon France; ^27^ Pediatric pneumology and allergology unit Hôpitaux pédiatriques de Nice CHU‐Lenval Nice France; ^28^ Pediatric Nutrition and Gastroenterology, Trousseau Hospital, AP‐HP Sorbonne Université Paris France; ^29^ Sorbonne Université, Inserm, Nutrition et obésités: approches systémiques Paris France; ^30^ Gut, Liver & Microbiome Research (GLIMMER) FHU Paris France; ^31^ INRAE, UMR1319 Micalis, AgroParisTech Jouy‐en‐Josas France

**Keywords:** children, dose, emergency, FPIES, OFC, ondansetron, rehydration

## Abstract

Food Protein‐Induced Enterocolitis Syndrome (FPIES) is a non‐IgE‐mediated food allergy marked by delayed vomiting and potential hypovolemic shock. This review by the French FPIES working group (SFA, GFRUP, Sp2A, SFP) analyzes recent literature on oral food challenges (OFC) adverse reactions and proposes a standardized protocol for OFC and emergency management. Early consensus OFC protocols from 2017 showed limitations—mainly short intervals between doses during OFC and high hospitalization burdens—leading to inconsistent practices. This narrative review of 2017–2025 studies shows that a single 25% age‐appropriate portion generally suffices to trigger symptoms, typically within 1–4 h. This approach balances safety and feasibility while reducing hospital time. As part of the French FPIES monitoring register, and to easily apply this portion, the serving size for each food has been established with specialized dieticians. According to severity, acute reactions require prompt rehydration and ondansetron. Oral or sublingual ondansetron, effective for mild to moderate reactions, can limit dehydration and hospital transfers. In a hospital setting, oral rehydration is indicated in mild reactions, whereas vascular hydration (10 mL/kg over 5–20 min) is required for moderate to severe reactions. Corticosteroids may be added in severe cases. Adrenaline is unnecessary unless associated IgE‐mediated symptoms are observed. The working group also proposes including oral rehydration solution and ondansetron in every home emergency kit. These proposals provide a national consensus on the management of FPIES by standardizing French practice from the OFC to the acute management of allergic reaction both in hospitals and at home.

**Key message**

We provide a narrative review of the existing literature on oral food challenges (OFC) in FPIES. Based on this review and our collective experience (allergists, pediatric gastroenterologists, emergency doctors, and dieticians), we propose practical and context‐adapted propositions, particularly regarding emergency care in the hospital and outpatient settings. We also provide reference values for serving sizes according to the patient's age, facilitating the prescription of OFC. Our overarching goal is to promote the harmonization of FPIES practices, ensuring safer, more consistent, and accessible care for patients and families.

## 1 INTRODUCTION

Food protein‐induced enterocolitis syndrome (FPIES) is a non‐IgE‐mediated food allergy that presents with repeated vomiting 1–4 h after ingestion of the trigger food, often with lethargy, pallor, and sometimes diarrhea within 24 h. Symptoms resolve quickly after food elimination. Severity ranges from mild (with 1–2 episodes of vomiting without lethargy) to severe reactions (≥3 episodes of vomiting with severe lethargy and/or hemodynamic impairment, potentially progressing to hypovolemic shock and malaise). Diagnosis and treatment follow the 2017 international consensus,^1^ for which the algorithm for diagnosis and treatment was updated in 2024.^2^

The oral food challenge (OFC) helps confirm diagnosis or tolerance acquisition, but protocols from the 2017 international consensus and before had limits. Since 2017, new OFC protocols were subsequently published and will be described in this paper.

The FPIES French working group comprises the SFA (French Allergy Society), supported by national partners (GFRUP: French‐speaking Pediatric Resuscitation and Emergency Group, and Sp2A: French Society of Pediatric Pneumology and Allergology of the SFP: French Pediatric Society), and aims to standardize OFC protocol for FPIES in France, with reference values for serving sizes according to the patient's age, and to propose acute reaction management in hospital and outpatient settings.

## 2 METHODOLOGY

Following a PRISMA‐inspired approach, we looked for all published FPIES‐specific OFC protocols (2017–2025) using PubMed® ((oral food challenge[Title/Abstract]) OR (OFC[Title/Abstract])) AND ((FPIES[Title/Abstract]) OR (food protein induced enterocolitis syndrome[Title/Abstract])). The last update of the search, made on 27 April 2025, identified 105 articles on PubMed®. We have manually added three other publications found from citation searching. After a first screening step based on titles and abstracts, and then exclusion of articles without OFC description in hospital settings (*n* = 19), articles involving adult patients (*n* = 2), case reports (n = 19), reviews (*n* = 20), practical guidelines (*n* = 5), surveys (*n* = 2), comments (*n* = 4), articles not related to FPIES (*n* = 2), unavailable articles (*n* = 3), and/or articles not in English (*n* = 2), we retained a total of 31 references focusing on OFC modalities in children in a hospital setting which are described in the “State of art for OFC modalities in FPIES”. The rest of this narrative review is less systematic, examining reports of oral or intravenous hydration, ondansetron, corticosteroids, and emergency kits across all publications identified through PubMed® and citations related to FPIES in children, both before and after 2017.

## 3 ORAL FOOD CHALLENGES

### 3.1 Current international guidelines

The 2017 international consensus proposed a 3‐dose FPIES‐specific OFC followed by a 4–6 h‐monitoring session with positivity criteria (Table 1).^1^ A 2‐dose protocol was also proposed for patients with a history of severe reactions, consisting of a “very low dose” of protein followed by 2–3 h of observation; if no reaction occurs, the patient ingests a full age‐appropriate portion and is monitored for 4 more hours.^1^

**TABLE 1:** Old and new OFC protocols.

2017 international consensus	New OFC protocol (FPIES French working group)
**Doses:** 0.06–0.6 g of protein/kg of the child's body weight, (average 0.3 g of protein/kg) divided into 3 equal parts (max 3 g of protein or 10 g of food or 100 mL of liquid)	**Eligibility criteria:** From 12 months of age No reaction in the last 12–18 months Regardless the patient's prior reaction severity **Prerequisite: check for associated IgE‐sensitization** (native food skin prick‐test) **OFC: dose and place** **Step 1:** 25% of an age‐appropriate serving size, during day hospitalization **Step 2:** 50% of an age‐appropriate serving size, at home **Step 3:** 100% of an age‐appropriate serving size, at home
**Duration** 30 min + monitoring for 4 to 6 h	**Duration** 3 consecutive or non‐consecutive days, in the morning or at lunch Monitoring for at least 4 h at step 1 in hospital (depending of history) and 4 h at steps 2 and 3 by parents
**Specific situations:** **IgE sensitization**: gradual progression of doses according to an OFC protocol adapted to an IgE‐mediated food allergy followed by monitoring for 6 h **History of severe reaction**: start at a lower dose and monitor for a longer time	**Specific situations:** **Atypical FPIES** (positive skin prick‐test or positive specific IgE) Increasing doses up to a cumulative dose corresponding to a full serving size according to the IgE‐mediated allergy protocol During day hospitalization Monitoring for at least 4 h at hospital **History of severe reaction:** lower first dose (e.g., 12.5%), then 25% at H2
**OFC positivity criteria:** Major criterion: vomiting within 1–4 h after OFC. + ≥ 2 minor criteria^a^: Lethargy Pallor Diarrhea (5–10 h after food ingestion) Hypotension Hypothermia Increased neutrophil count of ≥1500 neutrophils above the baseline count (if available).	

^a^ Could be absent if rapid use of ondansetron.

The 2024 update did not detail the OFC modalities.^2^

Both the 2017 international consensus and 2024 update recommend waiting at least 12–18 months after the last reaction before a supervised hospital OFC. The 2024 update evokes home OFC if there is no history of severe reaction, no sensitization, children older than the average age of resolution, and guardians comfortable with a home challenge.

### 3.2 State of art for OFC modalities in FPIES and related adverse events since 2017

#### 3.2.1 Dosing strategy

The 3‐dose protocol from the 2017 international consensus has limitations. As FPIES symptoms occur 1–4 h after ingestion, a 30‐min interval is insufficient to assess reactivity, making this protocol equivalent to a full‐dose challenge with the potential higher risk of severe reactions. The main limitation of the 2‐dose protocol is the duration of hospitalization due to the length of monitoring. Due to feasibility issues, neither protocol has gained wide acceptance.

A 2019 AAAAI (American Academy of Allergy Asthma and Immunology) survey of 132 allergists showed that although 95.5% performed OFC for IgE‐mediated allergies, only 53.8% performed OFC for FPIES, and 32.3% followed the 3‐dose 2017 international consensus protocol.^3^ A 2019 French survey (*n* = 92) also found variability in OFCs, with 21.7% of the practitioners used age‐dependent dosing, while 44% used a single‐dose OFC.^4^ These findings highlight the need for a standardized OFC protocol.

Among the total of 31 publications that reported details on OFC alongside other outcomes (Table 2), we identified 7 publications in which OFC was the main objective. We found that OFC protocols show substantial variability, with dosages expressed either as grams of protein per kilogram of body weight or as a percentage of the normal or age‐appropriate intake. However, portion sizes remain poorly defined, and administration may occur over one or multiple days.

**TABLE 2:** OFC descriptions since 2017.

Studied period and country	Mean or median age at OFC (months)	Cohort size (number of OFC)	Offending food	Number of doses the 1st day	Total number of days for challenge	Age‐dependent dose	Weight‐dependent dose	Dosing in brief	Previous severity‐dependent dose	Observation duration after last dose (hours)	Venous access?	References
2023 Italy	N/A	164	Several	3	1	No	Yes	3 equal portions over 30 min, 0.3 g protein/kg of body weight	No	6	Always	Carucci 2025 ^5^
2017–2022 Spain/Italy	64	267	Several	2	1	Yes	No	Protocol 1: H0 10% and H2 90% of age‐appropriate portions	No	4	Always	Argiz 2024^a^
				3	1	Yes	No	Protocol 2: H0 10%, H0.75 30%, H2.75 60% of age‐appropriate portions	No	4	Always	
				3	1	Yes	Yes	Protocol 3: 0.3 g protein/kg body weight (maximum 3 g in total) in 3 equal portions every 45 min; 4 h later, optional 4th dose consisting of an age‐appropriate portion	No	4	Always	
				1	2	Yes	No	Protocol 4: Day 1; 25% of an age‐appropriate dose; Day 2: 100% of an age‐appropriate portion	No	4	Always	
2020–2024 Japan	10	40	Solid food (egg, fish, soybean)	N/A	1	N/A	N/A	Total dose divided, at intervals of 30–60 min at the discretion of the physician for each case	N/A	24	N/A	Nagata 2024^7^
2020–2023 Japan	10	18	Several	1	1	N/A	N/A	At the discretion of the allergist based on patient history and other pertinent factors	Possible	N/A	N/A	Kunigami 2024^8^
2020–2022 Japan	N/A	225	Several	1	1–3	Yes	No	Single dose, according to age or previous severity, or at the dose that previously elicited symptoms	Yes	N/A	N/A	Hayashi 2024^9^
2018–2023 Japan	14	35	Hen's egg	1	At least 2	Yes	No	Single dose in hospital, then increase at home (50% of the final amount 3 times, followed by 75% of the final amount 3 times, followed by completion of the total amount 3 times)	Possible	N/A	N/A	Kajita 2023^10^
2020–2022 Japan	12	53	Several (egg, milk, wheat, soy)	1	1	N/A	N/A	Single dose, based on the patient's history and other factors	Possible	N/A	N/A	Kunigami 2023^11^
2010–2022 USA	1–2 (36%)	108 (185 OFC)	Several (milk, wheat, soy, rice, oat, egg, peanut, other)	1–2	1	N/A	Yes	Single‐serving portion, 0.3 g food protein/kg body weight (maximum 3 g food protein)	N/A	4	At the physician's discretion.	Patel 2023^12^
2014–2019 Japan	21	23	Several (soy, hen's egg, milk, wheat, rice, fish)	1	4	Yes	No	Single portion a day, at hospital: 1/50, 1/10, 1/2, full dose	Yes	4	Always	Nishimura 2022^a^
2010–2021 Japan	N/A	50	Several	3	1	Yes	No	3‐dose escalation protocol: (1) starting dose similar to the eliciting dose during the last symptomatic episode, (2) intermediate dose, (3) age‐appropriate serving size (maximum dose at the provider's discretion). Minimum interval of 4 h	N/A	4	N/A	Hayano 2022^14^
2019–2021 Japan	11	12	Hen's egg	1	1	No	No	Dose that previously elicited symptoms	No	N/A	N/A	Makita 2022^15^
2014–2020 France	24	179	Several	1 or several	1	Yes	Yes	Appropriate age‐serving size given in a single portion, or in 2 to 3 equal doses administered over 30 min	Yes	4	Always	Lemoine 2022^16^
2014–2020 Germany	17	100 (116)	Several (cow's milk, fish, vegetables, meat, grains, egg, others)	3	1	No	Yes	Appropriate age‐serving size in 3 doses every 90 min (10%, 30%, 60%)	N/A	N/A	N/A	Lange 2022^17^
2014–2018 Poland	4	55	Cow's milk	8	7	No	No	Hospital: lip test with a drop of milk, followed with gradually higher doses of milk every 15 min (1, 2, 5, 10, 20, 50, and 100 mL). Then, one meal with milk per day for the next 6 days at home		4–8	Always at the first OFC; then, only if positive milk specific IgE	Bulsa 2021^18^
2015–2019 Turkey	28	59	Several	3	1	No	Yes	0.06–0.6 g of food protein per kilogram of body weight (usually 0.3 g of protein per kilogram of body weight; to a maximum of 10 g of protein) in 3 equal doses over 45 min	N/A	4	N/A	Ocak 2021^19^
2017–2019 Italy	6	54	Several	2	1	No	Yes	0.3 g of food protein per kilogram of body weight: H0: 25% of the full dose; H4: 75% of the full dose; full dose	No	4	N/A	Ballini 2021^20^
2011–2020 Italy	27	91 (222 OFC)	Several	1	1	Yes	No	Single dose, ≥ full serving size for age, within 30 min	No	4	Only if necessary	Miceli Sopo 2021 ^rgiz 2024^ ^21^
2000–2020 Spain/Italy	9	70	Fish	1	3	Yes	No	Appropriate age‐serving size D1: 25%, D3: 50%, D5: 100%	No	2	N/A	Infante 2021^22^
2010–2018 USA	18.5	46 (88 OFC)	Various	3 to 4	1	Yes	Yes	Initial doses: 0.03–0.6 g of food protein per kilogram of body weight in 3 equal doses over 30 min + an age‐appropriate serving size 3 h later at the discretion of the physician	Possible	3	Not always	Guenther 2020^a^
Period: N/A Italy	5.3	11	Donkey's milk	4	1	No	Yes	0.06–0.6 g of food protein per kilogram of body weight (max 3–6 g of proteins, or 10–20 g of total food weight) in 3 portions over 45–60 min + an age‐appropriate serving size 2–3 h later	Yes	2–3	Always	Sarti 2019^24^
2010–2017 Greece	45	72 (69 OFC)	Various	4	1	Yes	Yes	0.1–0.3 g protein/kg body weight in 3 equal doses every 30 min; H2: full age‐appropriate dose	Possible	4–6	Always	Xepapadaki 2019^25^
2016–2018 Italy	N/A	48 (54 OFC)	Various	2	1	No	Yes	0.3 g protein/kg body weight, 25% at H0, and 75% at H4	Yes	4	Always	Barni 2019^a^
1996–2015 Spain	36	40 (43 OFC)	Fish	4	1	Yes	No	Appropriate age‐serving size Method 1: 12.5%, 25%, 50%, 100% every 30 min	No	2	N/A	Infante 2019^a^
2015–2018 Spain	60	40 (32 OFC)	Fish	1	3	Yes	No	Appropriate age‐serving size Method 2: D1: 25%, D3: 50%, D5: 100%	No	4	N/A	
2013–2017 Greece	N/A	78 (99 OFC)	Various	3	1	No	Yes	0.06 to 0.6 g of food protein/kg in 3 equal doses over 45 min. 2–3 h later, full dose	No	4	At the physician's discretion. Always if previous severe reaction	Douros 2019^28^
2014–2017 USA	37	119 (169 OFC)	Various	1	9 to 12	Yes	No	1/3 of serving size at hospital, gradually increase at home, every 3 days, over 9–12 days	No	4	At the physician's discretion	Wang 2019^a^
2015–2017 Japan	11	7 (8 OFC)	Cow's milk	1	3	No	Yes	1 mL/kg (up to 10 mL) at D1. D2: 5 mL/kg (up to 50 mL). D3: normal volume (up to 200 mL)	No	N/A	N/A	Shimomura 2018^30^
2013–2016 Italy/USA	N/A	16 (8 OFC)	Various	3	1	No	Yes	0.3 g of food protein per kilogram of body weight in 3 equal doses over 30 min (max 3 g of protein or 10 g of total food)	No	6	Always	Pecora 2017^31^
2013–2016 Japan	14.5	4	Cow's milk	1	2	No	Yes	5 mL/kg (up to 50 mL) on first day; next day: normal age‐appropriate dose	Yes	N/A	Always	Kimura 2017^32^
Period: N/A Italy/Australia	24	66	Several	1	1	No	No	Single oral dose, at least 3 g of food protein (for rice, 1–2 g of rice protein)	N/A	N/A	Always in Sydney, but not in Rome	Miceli Sopo 2017^33^
Period: N/A USA	N/A	38	Cow's milk	3	1	No	Yes	3 equal portions over an hour, 0.6 g food protein/kg of body weight	Yes	4–6	Always	Caubet 2017^34^
2008–2013 Spain	37	81	Several	3–7	1	No	Yes	0.3 g of food protein per kilogram of body weight in 3 equal doses every 90 min (max 3 g of protein) Milk: 7 consecutive doses every 90 min	Yes	24	Always	Vazquez‐Ortiz 2017 ^35^

Abbreviations: N/A, data not available; OFC, oral food challenge.

^a^ OFC as primary outcome.

Vazquez‐Ortiz et al.^35^ administered 0.3 g of protein/kg in 3 doses 90 min apart, except for cow's milk, which was administered in 7 consecutive doses 90 min apart. Guenther et al.,^23^ Sarti et al.,^24^ and Douros et al.^28^ also described OFCs using the three‐dose protocol over 30–45 min inspired by the 2017 international consensus, followed by an additional age‐adapted portion 3–4 h later.

In Lemoine et al.'s study^16^ and in Lange et al.'s study,^17^ children received an appropriate age‐serving size in 3 doses every 30–90 min.

Barni et al.^26^ and Ballini et al.^20^ proposed a protocol with 25% of a usual serving size for age followed by 4 h of monitoring, then 75% of a serving size with another 4‐h observation on the same day.

Wang et al.^29^ combined a hospital‐administered dose (1/3 of a serving size) with gradual home dose increases of one additional third every 3 days, over 9 days. If the escalation was over 12 days, patients received three‐quarters of a serving size on days 7–9 and 100% of the serving size on days 10–12.

Infante et al.^22^, ^27^ retrospectively compared two OFC methods in patients with FPIES to fish. Method 1 used increasing doses (1/8 + 1/4 + 1/2 + the rest of the portion for the age) at 30‐min intervals over one day, while Method 2 administered 25% of a usual portion on day 1, 50% at least 48 h later, then a full‐age appropriate dose after another 48 h.

Miceli Sopo et al.^21^ retrospectively analyzed OFCs over 9 years, challenging pediatric patients with a single full age‐appropriate dose in 1 day.

Nishimura et al.^13^ used a 4‐day OFC protocol with a single portion a day in the hospital setting: 1/50 at day 1, 1/10 at day 2, ½ at day 3, and full dose at day 4.

Argiz et al.^6^ prospectively compared 4 OFC protocols in Spain and Italy (Table 2): three one‐day OFC protocols, with 2 to 3 doses (protocol 1: H0 10%, H2 90% of age‐appropriate portions; protocol 2: H0 10%, H0.75 30%, H2.75 60% of age‐appropriate portions; protocol 3: 0.3 g protein/kg body weight (maximum 3 g in total) in 3 equal portions every 45 min), and a 2‐day OFC protocol (protocol 4: 25% of a portion on day 1, 100% on day 2). At the end, the cumulative dose was slightly lower in protocol 4 (2.6 vs. 5.4 g of protein, *p* = .048).

Split dosing at short intervals (as in the 3‐dose protocol over 30 min) appears to offer limited benefit, except possibly in patients who are already sensitized. The overall phenotype switch rate is approximately 1.1%, but increases to 13% among sensitized patients and may reach up to 29% of patients with FPIES sensitized to cow's milk. Anaphylaxis has been reported in 5.6% of sensitized patients with FPIES.^36^ Notably, 3 out of 10 studies published up to 2023 that reported a phenotype switch from FPIES to IgE‐mediated food allergy indicated that they adapted their protocol by using multiple incremental doses in cases of IgE sensitization.^17^, ^25^, ^37^

New trends favor home‐based OFCs for low‐risk cases.^38^ In Canadian experience with an egg ladder, children with mild to moderate FPIES tolerated cooked egg within 7 months on average.^39^ Although no randomized trials compare home versus hospital reintroduction, home protocols may speed recovery but require further study. OFCs conducted at home from the outset are outside the scope of the present review.

#### 3.2.2 Observation period

The observation period after the last dose usually ranges from 4 to 6 h,^5^, ^6^, ^13^, ^14^, ^16^, ^18^, ^19^, ^20^, ^21^, ^25^, ^26^, ^27^, ^28^, ^29^, ^31^, ^34^ sometimes 2–3 h,^23^, ^24^, ^27^, ^40^ and 24 h in rare cases.^7^, ^35^

In Wang et al.'s study,^29^ reactions during OFC at hospital were mainly vomiting (*n* = 17), then diarrhea (*n* = 3). Oral rehydration was possible in 3 patients out of 17. Arterial hypotension was noticed in 2 patients. In 5 cases, patients were hospitalized after the OFC due to symptom persistence. At home, none of these reactions were severe. Indeed, they were mostly delayed diarrhea (*n* = 13) and vomiting (*n* = 3). One single reaction led to a consultation in the emergency room without having to resort to hospitalization.

In Infante et al.'s study,^27^ the severity of adverse reactions in method 1 (several doses in 1 day) was, in order of frequency, moderate (41.9%), severe (39.5%), or mild (18.6%) while it was in method 2 (3‐day protocol with single daily dose), in order of frequency, mild (68.8%), moderate (18.8%), or severe (12.5%) (*p* < .001 between methods 1 and 2). The relative risk of moderate symptoms was 2.2‐fold higher (95% CI: 1.0–5.0) and the risk of severe symptoms was 3.2‐fold higher (95% CI: 1.2–8.5), in the one‐day protocol compared with the 3‐day protocol. With similar OFC protocols, Argiz et al.^6^ evidenced that a one‐day OFC protocols (protocols 1 to 3) caused more severe reactions than the two‐day OFCs (protocol 4) (one‐day OFC: mild 13%, moderate 58%, severe 30%; two‐day OFC: mild 55%, moderate 41%, severe 5%; p < .001; OR = 10.5, 95% CI: 1.28–87.1, *p* = .03). No children required intensive care. The cumulative dose was not linked to severity. Previous severe reactions did not increase OFC risk (*p* = .96).

In Barni et al.'s protocol,^26^ after the 25% of 0.3 g of protein/kg body weight, symptoms were severe in 9 cases (47.3%), moderate in 6 (31.6%) and mild in 4 (21.1%). Only two patients (10.5%) received no treatment, and none required transfer to intensive care. In Italy,^20^ with a similar protocol, severe reactions occurred in 5 OFCs (35.7%), moderate in 3 OFCs (21.4%), and mild in 6 OFCs (42.8%). Five patients (42.8%) did not require any treatment. With the protocol from 2017 international consensus, severity of reaction was mild in 12 (18.2%), moderate in 18 (27.3%), and severe in 36 OFCs (54.5%) in Turkey,^19^ and mild‐to‐moderate in 4 (50%), severe or very severe in 4 (50%) in Italy, with a correlation with CRP levels.^31^

In Xepapadaki's study,^25^ two patients presented with pallor and hypotension. It was not specified whether these reactions occurred with the initial doses or after the full age‐appropriate dose.

Miceli Sopo et al.^21^ reported mostly mild or moderate reactions (22 each out of 48), with a single full‐dose protocol. Only 4 reactions (8.3%) were severe: 2 occurred in children younger than 12 months and 2 in children aged 4–5 years, including 2 cases involving processed foods (baked egg and parmesan). Only one of these four patients had a history of a prior severe reaction. Two of the four severe cases involved atypical FPIES. In 33.3% of failed OFCs (16/48), no treatment was required. The authors reported that the risk of severe reactions was approximately four times higher in sensitized patients compared with non‐sensitized patients, although this difference was not statistically significant (*p* = .3). Dose was not identified as a risk factor for severity; however, no control group was included.

In the Japanese study,^13^ none of the small number of patients who reacted had a severe reaction with less than 1/10 of a serving size.

It has been hypothesized that early administration of ondansetron may prevent progression toward a more severe adverse reaction.^1^

#### 3.2.3 Peripheral intravenous access during OFC

According to the current narrative review (Table 2), 13 of the 31 reviewed studies reported a systematic placement of a peripheral intravenous (IV) access before OFC.^5^, ^6^, ^13^, ^16^, ^18^, ^24^, ^25^, ^26^, ^31^, ^32^, ^33^, ^34^, ^35^ In 5 studies, IV access was at the physician's discretion,^21^, ^23^, ^28^, ^29^, ^33^ and 14 did not mention whether IV access was systematically placed or not.^7^, ^8^, ^9^, ^10^, ^11^, ^12^, ^14^, ^15^, ^17^, ^19^, ^20^, ^22^, ^27^, ^30^

Some arguments against systematic peripheral IV access before OFC are summarized in a perspective paper by Ruffner et al. In brief, only 10% to 55% of patients experience reactions during OFC, and fewer than 20% need IV fluids.^41^ Moreover, despite local analgesia, IV access could cause pain, fear and anxiety in children, as well as distress for parents.^42^, ^43^ Finally, peripheral IV access implies a day‐unit hospitalization for supervised OFC. However, Ford et al. stated arguments in favor of systematic IV access before carrying out an FPIES‐specific OFC are stronger.^44^ First of all, no predictive factor of severe reaction has been identified. Indeed, hypotension occurs in 5%–77% of acute FPIES cases.^45^ Moreover, prior moderate to severe reaction did not predict severity in Wang's study.^29^ However, risk increases if there has been a previous severe reaction and with a recent reaction.^16^ Younger children more often need IV treatment.^46^ Finally, and above all, early IV placement is warranted due to dehydration and vasoplegia risks during reactions.

### 3.3 Proposals from the FPIES French working group for OFC modalities

OFC is indicated for purposes of diagnosis or to confirm recovery. Indeed, performing an OFC within 12 months of the initial reaction increases the risk of failure.^16^ The median age for tolerance acquisition across all foods is 2.5 years, but 6.1 years for fish in France.^16^ It was agreed to perform an OFC 12–18 months after the last reaction, and to repeat it only after age 5 in cases of fish allergy. However, an earlier OFC may be considered to assess tolerance to alternative fish.^47^

The OFC modalities proposed here by the FPIES French working group consisting of three steps (the first step in the hospital, followed by second and third steps at home) are indicated from 12 months of age, provided there has been no reaction in the preceding 12–18 months, regardless of the patient's prior reaction severity. Before performing the OFC, even though the risk of associated sensitization and phenotype switch are low (below 10% and 1.2%, respectively),^36^ we recommend confirming the absence of IgE‐mediated sensitization using an immediate‐reading skin prick test, expected to be negative (Table 1). Recent specific IgE results and/or a skin prick test performed within the previous 3 months may obviate the need for repeat skin prick testing on the day of the OFC. The skin prick test is a simple, rapid, and low‐cost procedure, requiring only trained healthcare professionals and minimal materials. This test is generally well tolerated by patients and specific for excluding IgE‐mediated sensitization.^48^ The relevance of this systematic testing will be specifically evaluated in our national prospective FPIES monitoring register (NCT05528900, ClinicalTrials.gov). Further recommendations are needed to determine whether screening for associated sensitization should be restricted to selected patients, such as those with atopic dermatitis.

We recommend that a functional peripheral intravenous line be systematically placed before starting the first OFC step in a hospital setting.

Based on the literature reviewed here (period: 2017–2025, children only), on a previous review of Bird et al. (1978–2020)^49^ and on the recent systematic review of Ibrahim et al. (more than 3500 OFCs performed in both children and adults, from 1978 to 2024),^50^ we concluded that a single dose corresponding to 25% of the normal age‐appropriate portion is in most cases sufficient to trigger a reaction in most cases, usually within 2–3 h after ingestion, and that no severe reaction is expected at subsequent feed doses if the initial one is tolerated. The classic FPIES protocol involves administering 25% of an age‐appropriate portion during day hospitalization, followed by at least 4 h of monitoring, depending on the previous reaction. If no reaction occurs, parents receive oral and written information explaining the FPIES home reintroduction protocol and the procedure to follow in case of an allergic reaction (Appendix S1). Practitioners should ensure that parents fully understand the home protocol before hospital discharge. If parents are confident, the patient will consume 50% of an age‐appropriate portion at home on another day (either the next day or a different day). Finally, on a third day (which may or may not be a consecutive day), a full age‐appropriate serving size is given. Home administration should preferably be in the morning, no later than midday, to allow parental supervision for at least 4 h. In the rare cases where the physician considers that parents are not able to safely carry out home reintroduction, reintroduction may be proposed over 1 day (two doses administered at least 2 h apart, or longer depending on previous reaction latency) or over 2 days in a hospital setting (day hospital or outpatient consultation, at the allergist's discretion), under medical supervision.

If there is a severe reaction history requiring intensive care, the OFC may include several hospital doses. The first dose should be lower, at the allergist's discretion (e.g., 12.5% of the serving size or less), with 2 h of monitoring before the next dose, up to 25% of an age‐appropriate portion. The following doses (50% then 100%) may also be given during two other day hospitalizations, especially if the family is concerned about performing it at home without supervision.

In cases of IgE‐mediated sensitization (atypical FPIES), a 1 day more gradual dose‐escalation protocol corresponding to the IgE‐mediated OFC protocol should be applied, i.e., increasing doses up to a cumulative amount equivalent to one full serving size according to the IgE‐mediated allergy protocol,^51^ followed by at least 4 h of monitoring.

Table 3 lists age‐appropriate portion sizes for major foods involved in FPIES and their protein equivalents. It was developed with the Allergodiet group, composed of dietitians specializing in allergies, to standardize OFC procedures, in a day‐unit hospital setting.

**TABLE 3:** Usual portion sizes according to age.

Food	Average protein content (g/100 g)	Average quantity per dose according to age	25% of the age‐appropriate serving size	Protein amount (in g)
*Milk and dairy products*				
Semi‐skimmed cow's milk (or powdered infant milk before 12 months)	3.3%	4–6 months: 210 mL	50 mL	1.7 (0.6)
		6–12 months: 240 mL	60 mL	2 (0.7)
		12–24 months: 270 mL	70 mL	2.3
		Over 3 years: 300 mL	75 mL	2.5
Yogurt	3.8%	125 g	30 g	1.1
*Egg*				
Whole egg	13.5%	Before 12 months: 10 g	2.5 g	0.3
		1–2 years: 20 g	5 g	0.7
		2–3 years: 30 g	7.5 g	1
		Over 3 years: 50 g (=1 egg)	12.5 g	1.7
*Meat*				
Cooked beef/veal and chicken/turkey	27.2%	6–12 months: 10 g	2.5 g	0.7
		1–2 years: 20 g	5 g	1.4
		2–3 years: 30 g	7.5 g	2
		3–6 years: 50 g	12.5 g	3.4
		6–12 years: 70 g	17.5 g	4.8
		>12 years: 100 g	25 g	6.8
*Fish and seafood*				
Cooked fish	24%	6–12 months: 10 g	2.5 g	0.6
		12–24 months: 20 g	5 g	1.2
		24–36 months: 30 g	7.5 g	1.8
		3–6 years: 50 g	12.5 g	3
		6–12 years: 70 g	17.5 g	4.2
		>12 years: 100 g	25 g	6
Cooked shrimp/crab/mussels/scallops	19%	50 g or more	12.5 g	2.4
*Legumes*				
Cooked pea	5.2%	6 months–12 years: 100 g	25 g	1.3
		>12 years: 150–200 g	37.5–50 g	2.0–2.6
Soy drink^a^	3.9%	100 mL	25 mL	1.1
Soy (dessert)^a^	4.6%	100 g	25 g	1.1
Peanut	22.0%	15 g	4 g	0.9
*Wheat*				
Cooked pasta or semolina	5.8/6.1%	6–9 months: 25 g	6.5 g	0.4
		9–12 months: 50 g	12.5 g	0.8
		1–3 years: 80 g	20 g	1.2
		3–6 years:120 g	30 g	1.8
		6–12 years: 170 g	42.5 g	2.6
		>12 years: 200–250 g	50–62.5 g	3.0–3.8
Baby cereals	13.6%	6–9 months; 20 g	5 g	0.7
		9–12 months: 25 g	6.3 g	0.9
Bread	9%	>12 months: up to 1 small 40 g slice	10 g	0.9
*Other starches*				
Cooked rice	3.1%	6–9 months: 25 g	6.5 g	0.2
		9–12 months: 50 g	12.5 g	0.4
		1–3 years: 75 g	20 g	0.6
		3 –6 years: 120 g	30 g	1.1
		6–12 years: 170 g	42.5 g	1.3
		>12 years: 200–250 g	50–62.5 g	1.6–1.9
Cooked corn: main course (or starter)	2.8%	6–9 months: 25 g	6.5 g	0.2
		9–12 months: 50 g	12.5 g	0.3
		1–3 years: 75 g	20 g	0.6
		3–6 years: 120 g (60 g)	30 g (15 g)	0.8 (0.4)
		6–12 years: 170 g (80 g)	42.5 g (20 g)	1.2 (0.4)
		>12 years: 200–250 g (100–150 g)	50–62.5 g (25–37.5 g)	1.4–1.8 (0.7–1.1)
Cooked potato/Sweet potato	1.8%/1.7%	6–9 months: 25 g	6.5 g	0.1
		9–12 months: 50 g	12.5 g	0.2
		1–3 years: 75 g	20 g	0.3
		3–6 years: 120 g	30 g	0.5
		6–12 years: 170 g	42.5 g	0.7
		>12 years: 200–250 g	50–62.5 g	0.9–1.1
*Vegetables and fruits*				
Cooked green beans	1.8%	6 months‐12 years: 100 g	40 g	0.7
		>12 years: 150–200 g	37.5–50 g	0.7–0.9
Raw apple or compote	0.3%	6 months‐12 years: 100 g	25 g	0.1
		>12 years: 150 g	37.5 g	0.1
Raw banana or compote	1.1%	6 months‐12 years: 100 g	25 g	0.3
		12 years: 150 g	37.5 g	0.4

^a^ Soy‐based products are not recommended in collective catering, whatever the population (ANSES, Referral n° 2022‐SA‐0221).

*Source:* High council for public health opinion on the revision of dietary guidelines for children aged 0–36 months and 3–17 years (30/06/2020); GEMRCN nutritional recommendations (weight tables by age group and by food in collective catering); Anses. 2025. Ciqual nutritional composition table for foods, https://doi.org/10.57745/RDMHWY.

In cases of particular comorbidities, the OFC protocol may be adjusted by practitioners depending on the nature of the comorbid conditions.

As part of a national prospective FPIES monitoring register (NCT05528900, ClinicalTrials.gov) which is already implemented in most French centers since 2023, we propose to evaluate this FPIES‐specific OFC protocol and safety with 25% of the normal age‐appropriate dose in day‐unit hospitalization.

## 4 MANAGEMENT OF AN ACUTE ALLERGIC REACTION IN A HOSPITAL SETTING

### 4.1 Current international guidelines

Treatment of an acute reaction depends on symptom severity (mild, moderate, severe) (Table 4) and the setting (hospital or home).^1^, ^2^

**TABLE 4:** Severity and management of FPIES acute reaction in the hospital setting.

Mild reaction	Moderate reaction	Severe reaction
1 to 2 vomiting no lethargy	≥ 3 vomiting + moderate lethargy	≥ 3 vomiting + severe lethargy or hypotonia or hemodynamic impairment
**Oral or sublingual ondansetron** (age ≥6 months) 8–15 kg: 2 mg 16–30 kg: 4 mg >30 kg: 8 mg to be renewed if oral ondansetron and vomiting within 10 min **or IV ondansetron** 0.15 mg/kg or same dosage as oral route (maximum 8 mg, age ≥6 months) Infusion over at least 15 min Then: **oral rehydration solution**: divided administration in small quantities 20 min after ondansetron	**Vascular hydration:** 10 mL/kg over 5–20 min with balanced isotonic solution (e.g., Isofundin®, Plasmalyte® or Ringer Lactate) or NaCl 0.9% To be renewed if necessary (up to 40–60 ml/kg during the first hour) + **IV ondansetron:** 0.15 mg/kg or same dosage as oral route (maximum 8 mg, age ≥6 months) Infusion over at least 15 min	**Vascular hydration:** 10 mL/kg over 5–20 min with balanced isotonic solution (Isofundin®, Plasmalyte® or Ringer Lactate) or NaCl 0.9% To be renewed if necessary (up to 40–60 mL/kg during the first hour) **+ IV ondansetron:** 0.15 mg/kg or same dosage as oral route (maximum 8 mg, age ≥6 months) Infusion over at least 15 min **+ IV corticosteroids: 1 mg/kg (max 60 mg)** ± methemoglobinemia monitoring ± correction of hydro‐electrolyte and/or acid–base disorders
*Minimum monitoring*: **2 h** after resolution of symptoms Check fluid tolerance before discharge.	*Minimum monitoring*: **4 h** after resolution of symptoms Check fluid tolerance before discharge. Hospitalization if persistent symptoms	*Minimum monitoring*: **4–6 h** after resolution of symptoms Check fluid tolerance before discharge **Intensive care unit hospitalization if poor hemodynamic tolerance**

Maintaining hydration and hemodynamic stability is crucial and may require oral rehydration for mild reactions or intravenous infusion, even volume expansion, for severe ones.^1^, ^2^

Use of ondansetron during hospital‐based reactions has been reported to reduce vomiting duration and prevent more severe symptoms. It can be given to children over 6 months old to limit persistent vomiting, pallor, and lethargy. The IV/IM dose is 0.15 mg/kg (maximum 8 mg).^1^, ^2^

Corticosteroid administration should be considered in severe reactions due to presumed cellular inflammation.^52^

Correction of hydro‐electrolyte or acid–base imbalances and methemoglobinemia may be required in severe cases.

Unlike IgE‐dependent food allergies at risk of anaphylaxis, adrenaline has no role in FPIES management; however, vasopressors may be needed for hypotension unresponsive to fluid resuscitation.

### 4.2 State of art for managing acute reaction in a hospital setting

#### 4.2.1 Oral or intravenous hydration

Hydration is a key component in managing acute FPIES reactions due to the risk of dehydration and hypovolemic shock, especially with repetitive vomiting. In brief, the need for rehydration during OFC varied across studies, from 8%^46^ to 100%.^53^, ^54^

In protocols resembling international consensus guidelines, the proportion of patients requiring IV fluids ranged from 10%^23^ to 58%,^35^ and up to 83% in the study by Ocak et al.^19^ In slower protocols extending over several days, intravenous normal saline boluses were administered in 5 of 8 cases with moderate reactions.^13^ Protocols starting with an initial dose ranging from 25% to one‐third of a serving size or one third of 0.3 g of protein per kilogram of body weight reported IV fluid resuscitation rates of 47%,^20^ 58%,^26^ or 82%.^29^ When a full serving was given as a single dose, IV fluids were required in 21% of failed OFCs.^12^ In contrast, gradual protocols similar to those used for IgE‐mediated food allergy were associated with IV hydration in 42% of children.^18^

It remains unclear whether IV rehydration was chosen based on severity criteria or simply preferred when venous access was available. Nevertheless, persistent vomiting despite the use of ondansetron is not a contraindication to oral rehydration.^55^

#### 4.2.2 Ondansetron

Ondansetron is a selective 5‐hydroxytryptamine3 (5HT3) receptor antagonist acting on enterochromaffin cells in the gastrointestinal mucosa, vagal afferents, and the area postrema of the brain stem, which forms the chemoreceptor trigger zone. It has strong central and peripheral antiemetic effects. According to the French National Authority for Health and Food and Drug Administration, indications of ondansetron concern the prevention and treatment of nausea and vomiting induced by chemotherapy and radiotherapy in adults and children (from 6 months of age depending on the indication), as well as postoperative nausea and vomiting in adults and children (from 1 month of age). Oral and sublingual ondansetron are increasingly used off‐label to effectively treat vomiting during acute gastroenteritis from 3 months of age.^56^, ^57^ In France, the recommended dosage is 5 mg/m^2^, or 0.15 mg/kg, administered 2–3 times per day, with a maximum single IV dose of 8 mg and a maximum adult daily dose of 32 mg.

In most studies on acute allergic reactions during OFC in FPIES, ondansetron is administered intravenously or intramuscularly.^21^, ^29^, ^33^, ^38^, ^58^, ^59^, ^60^, ^61^, ^62^, ^63^ However, the route of administration is often unspecified.^6^, ^18^, ^19^, ^25^, ^26^, ^64^, ^65^, ^66^, ^67^ The 2017 international consensus recommends IV or IM administration.

Nevertheless, the oral route also appears effective. According to Vila Sexto's experience, 6 of 7 patients improved after oral ondansetron (2 mg if 8–15 kg; 4 mg if 16–30 kg; 8 mg if >30 kg), with only one requiring oral rehydration.^55^ Wong et al. similarly reported two patients improving after sublingual ondansetron.^38^ In another small cohort (*n* = 6), oral ondansetron (4 mg if <16 kg; 8 mg if >16 kg) was evaluated as first‐line treatment during 44 OFCs for FPIES, resolving symptoms in 5 of 7 positive cases. Oral rehydration was needed once. In two others, symptoms stopped initially but recurred 30–45 min later, resolving after IV ondansetron.^68^ These studies support the effectiveness, ease of use, and good tolerance of oral ondansetron for mild acute FPIES symptoms. Recently, oral ondansetron has been increasingly used for this indication, including during hospital‐based OFCs.^69^, ^70^ Early administration has been reported to be sufficient to stop vomiting and avoid oral or IV rehydration.^55^, ^68^, ^69^, ^70^ No randomized controlled trial has compared parenteral (IV or IM) and oral/sublingual ondansetron. And the number of subjects is small and there are no controls.

From a pharmacokinetic perspective, in healthy subjects, IV ondansetron achieves faster and higher plasma concentrations (Cmax) than oral or IM administration (median time to Cmax: 7 min for IV and IM, 1 h for oral; Cmax: 80 ng/mL for IV 8 mg, 24 ng/mL for IM 4 mg, 31 ng/mL for oral 8 mg; no data available for sublingual administration).^71^

Regarding QT prolongation risk, existing data are reassuring. Long QT syndrome is rare—about 1 in 16,000 individuals—and ventricular arrhythmia due to ondansetron occurs in only 0.003% of treated patients.^72^ No significant ECG difference is observed before and after ondansetron in children.^73^, ^74^ Arrhythmias occur only with predisposing factors such as underlying heart disease, conduction disorders, or concomitant QT‐prolonging drugs.^72^, ^75^ Many experts therefore advise against routine ECG monitoring, especially since ondansetron is given as a single dose, unless cardiac risk factors are present.^72^, ^74^

In the French descriptive study, no QT prolongation was reported during patient monitoring.^68^

#### 4.2.3 Corticosteroids

Patients with severe symptoms are usually treated with methylprednisolone 1 mg/kg.^76^ Survey data show corticosteroids are used in 29–54% of acute reactions.^3^, ^77^, ^78^ According to several case series,^13^, ^19^, ^20^, ^21^, ^26^, ^54^, ^60^, ^64^, ^67^, ^70^, ^79^, ^80^, ^81^ corticosteroid use, by IV or oral route, varies widely—from 2% to 5%^26^, ^64^ to 94%^81^ of patients—depending on severity and/or age.^3^ However, no randomized study has yet demonstrated a true benefit of this treatment.

### 4.3 Proposals from the FPIES French working group and emergency specialists and resuscitators from GFRUP for the management of allergic reactions during OFC in a hospital environment

In the event of a mild reaction (Table 4), oral rehydration should be offered 20 min after oral or sublingual ondansetron.

For moderate to severe reactions, vascular hydration should be performed with a balanced isotonic solution (Isofundin®, Plasma‐Lyte® or Ringer Lactate) or, if unavailable, normal saline (NaCl 0.9%): IV bolus of 10 mL/kg over 5–20 min, repeated if necessary (up to 40–60 mL/kg during the first hour)^82^ (Table 4). This should be combined with oral, sublingual or IV ondansetron—preferably IV rather than IM, given the presence of venous access.

Regardless of severity, ondansetron should be administered as soon as vomiting begins to improve rehydration and prevent recurrence. The recommended dosage is 2 mg for 8–15 kg (over 6 months old), 4 mg for 15–30 kg, and 8 mg for >30 kg. Ondansetron may be given as syrup (Zophren® 4 mg/5 mL), sublingual film (SétofilmGé® 4 or 8 mg), or orodispersible tablet dissolved in water. IV dosage is similar: 0.15 mg/kg (maximum 8 mg). No pretherapeutic ECG is required unless there are cardiac risk factors such as familial long QT syndrome, underlying congenital cardiac conduction abnormalities, or other major cardiac diagnoses.

A single IV dose of methylprednisolone 1 mg/kg (maximum 60 mg)^76^ may be given in severe reactions.

Neither IM epinephrine nor antihistamines are indicated in acute FPIES, except in IgE‐mediated atypical forms.

Patients should remain under hospital supervision for at least 2 h (mild) to 4 h (moderate to severe) after symptom resolution. Hydro‐electrolyte imbalance, metabolic acidosis, or methemoglobinemia may be investigated and corrected if present. These may justify extended monitoring—up to 6 h—or short‐term hospitalization or intensive care depending on severity, until oral intake resumes. Severe or persistent forms despite initial treatment require intensive care.

Practitioners must ensure oral fluids are well tolerated before discharge.

## 5 EMERGENCY KIT AND TREATMENT PROTOCOL IN THE EVENT OF A REACTION AT HOME

### 5.1 Current international guidelines

Nowak‐Wegrzyn et al. classified mild reactions as those resolving with oral rehydration (breastfeeding or clear fluids) at home, and moderate to severe reactions as requiring urgent management (call to emergency services or visit to the emergency room) for rehydration and hemodynamic stabilization if needed.^1^ Epinephrine autoinjectors are not recommended except in concomitant IgE‐mediated food allergy with anaphylaxis risk. No clear guidance exists on the contents of emergency kits for patients and caregivers.

Since 2021, oral ondansetron at home has been recommended, with the option to repeat the dose if vomiting occurs within 10 min of the first.^83^ During the COVID‐19 pandemic, authors suggested not all patients need medical consultation after mild reactions without prior severe episodes. Oral rehydration should be attempted 20 min after vomiting, with or without ondansetron.

The 2024 update considers oral ondansetron (solution or disintegrating tablets) for non‐severe reactions at home, defined as fewer than three vomiting episodes, no moderate/severe lethargy, pallor, or hypothermia, no severe abdominal pain, possible oral rehydration, and no history of severe reactions.^2^

Experts recommend providing an emergency letter and action plan explaining FPIES, its particularities, and basic emergency care for medical practitioners.^2^, ^84^

### 5.2 State of art for emergency kits in the outpatient setting

Several surveys examined FPIES management, particularly the prescription of emergency kits for outpatient acute reactions.

A 2014 survey of AAAAI members (*n* = 470) reported that 84% provided emergency action plans, especially academic practitioners; 73% provided an emergency department letter explaining FPIES, and 21% prescribed epinephrine injectors, mainly when knowledge of FPIES was low.^85^

In France, two surveys were conducted: one among general private or mixed private‐hospital pediatricians (*n* = 375),^78^ and one among pediatric gastroenterologists and allergists (*n* = 92).^4^ Among general pediatricians, 63% reported limited or no knowledge of FPIES.^78^ Similar to American practitioners,^85^ 21% recommended adrenaline, 18% antihistamines, and 29% corticosteroids for acute FPIES in a hospital setting.^78^ Among pediatric specialists, oral rehydration solution and/or ondansetron were widely included in emergency kits (85% and 65%, respectively), but 25% and 15% still prescribed antihistamines and/or epinephrine.^4^ These findings highlight the need for continuous education and the importance of explanatory FPIES emergency letters, provided by the majority of the panel (84%).^4^

### 5.3 Proposals from the FPIES French working group and emergency specialists and resuscitators from GFRUP for the management of allergic reactions outside the hospital setting

#### 5.3.1 Composition of the emergency kit

The proposed emergency kit systematically includes an oral rehydration solution to be given at a rate of 15 mL every 10 min as well as ondansetron orally from the age of ≥6 months and ≥8 kg. The dosage is the same as that proposed for oral ondansetron in hospitalized patients, namely 2 mg between 8 and 15 kg (over 6 months old), 4 mg between 15 and 30 kg, and 8 mg for those over 30 kg. No pre‐therapeutic ECG is required, except if cardiac risk factors such as familial long QT syndrome, underlying congenital cardiac conduction abnormalities, or other major cardiac diagnoses.

We do not recommend the systematic inclusion of corticosteroids in the emergency kit, given the limited evidence of efficacy to date.

It should be recalled that adrenaline is not indicated in the treatment of acute classic FPIES. However, an adrenaline auto‐injector may be prescribed in cases of atypical FPIES (positive skin test and/or specific IgE to the food, with or without a known immediate IgE‐mediated allergic reaction), or if other IgE‐mediated food allergies are present. In such cases, an antihistamine should also be prescribed, and β_2_‐mimetics if asthma is associated.

#### 5.3.2 Action plan in the event of a reaction outside the hospital environment

**FIGURE 1:**
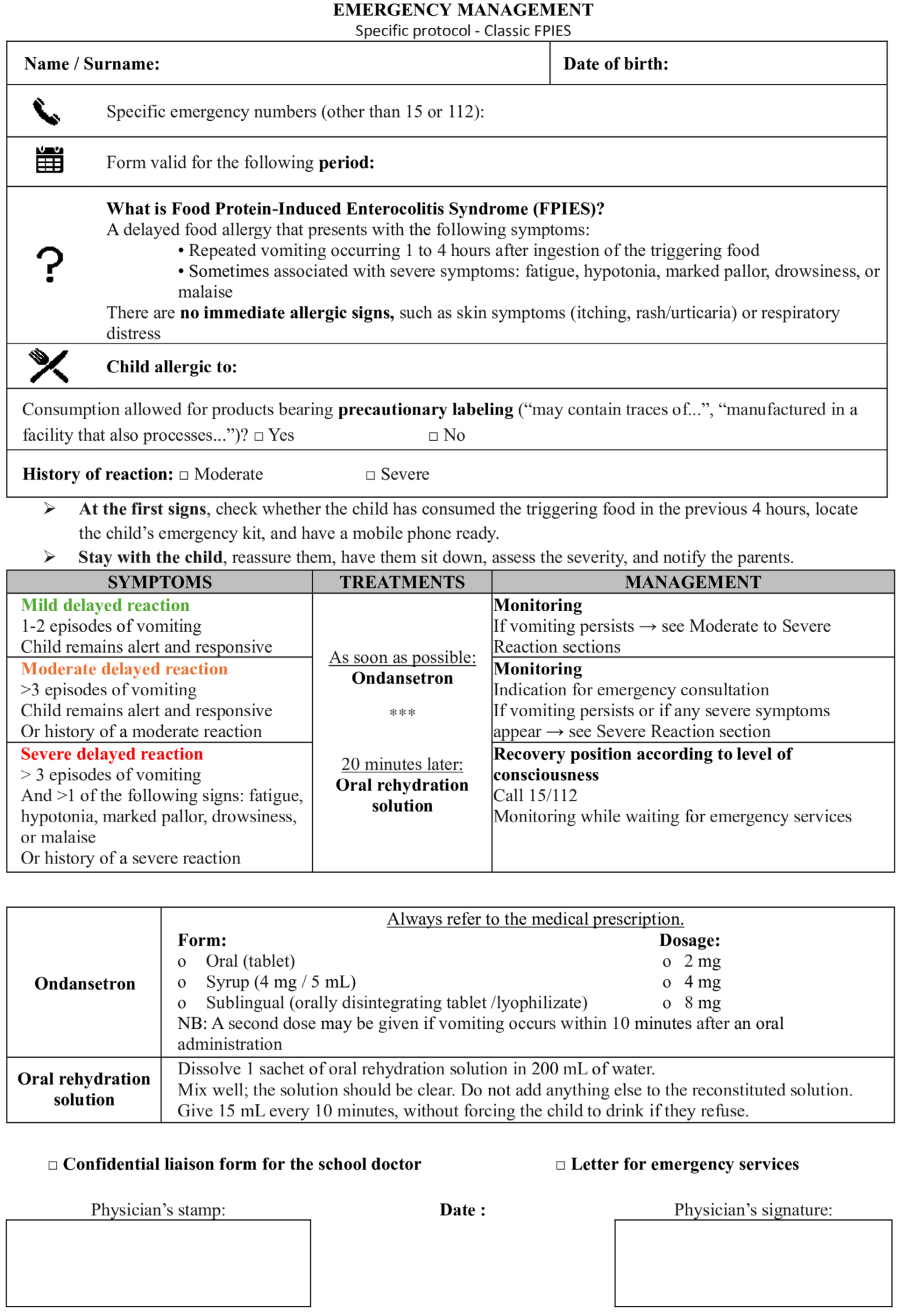
Management of FPIES acute reaction according to severity outside the hospital.

The action plan (Figure 1) must be clearly explained to families during follow‐up consultations. It is also important to explain it again after the OFC before returning home in case of a delayed reaction. The following protocol is based on the recommendations of Leonard et al.^83^

In the event of a mild reaction, ondansetron should be administered orally or sublingually as soon as possible, followed by oral rehydration 20 min later. A consultation in the emergency room is not systematically indicated.

In the event of a moderate reaction or a history of moderate reaction requiring intravenous rehydration in hospital, administer oral or sublingual ondansetron as soon as possible and attempt oral rehydration 20 min later while awaiting emergency consultation.

In the event of a severe reaction or a history of severe reaction requiring prolonged hospitalization for hydro‐electrolytic support, administer oral or sublingual ondansetron as soon as possible, call emergency medical services, and attempt oral rehydration while waiting for medical care.

If further vomiting occurs within 10 min after sublingual ondansetron, a new dose may be given orally. As vomiting is acute, it is not necessary to routinely repeat ondansetron 12 h later. However, if vomiting recurs during the 4‐ to 6‐h monitoring period, a new dose of IV or oral ondansetron may be given after a minimum interval of 4 h.

## 6 DISCUSSION

This narrative review from the French FPIES working group aimed to provide national consensus in the management of FPIES, ranging from the OFC to the acute management of allergic reaction. We recognize that the evidence supporting these proposals is primarily based on retrospective studies, with small sample sizes, heterogeneous protocols, and a lack of randomized controlled trials. Nevertheless, the 25% single‐dose strategy is aligned with international expert proposals^86^ and supported by a recent systematic review.^50^ In addition, ondansetron has demonstrated clinical effectiveness and good tolerance in the management of FPIES reactions, despite the absence of randomized controlled trials. Our work further contributes by providing a wide range of reference serving doses for different foods, along with a comprehensive management protocol for FPIES, covering both OFCs and reaction management. Importantly, the implementation of this protocol will allow for prospective evaluation in pediatric patients enrolled in a French FPIES pediatric registry.

## 7 CONCLUSION

The standardization of OFC as part of the first multicenter French‐speaking pediatric prospective cohort of acute FPIES will contribute to improving the diagnostic and therapeutic management of FPIES. The emergency home care protocol will also make it possible to optimize the level of safety in the event of a reaction during reintroduction at home and, ultimately, to validate a personalized action plan in case of a reaction adapted to this particular food allergy.

## AUTHOR CONTRIBUTIONS

**Virginie Jubin:** Conceptualization; writing – original draft; investigation; methodology; writing – review and editing; data curation; visualization; project administration; validation; formal analysis. **Pascale Dumond:** Conceptualization; writing – review and editing; methodology; investigation. **Grégoire Benoist:** Conceptualization; writing – review and editing; validation. **Flore Amat:** Writing – review and editing. **Harriet Corvol:** Writing – review and editing. **Laure Couderc‐Kohen:** Conceptualization; writing – review and editing. **Antoine Deschildre:** Conceptualization; writing – review and editing; supervision. **Angélique Doc:** Writing – review and editing; conceptualization; methodology. **Amandine Divaret‐Chauveau:** Writing – review and editing. **Kamal El Abd:** Writing – review and editing; conceptualization. **Agnès Linglart:** Writing – review and editing. **Carine Metz‐Favre:** Conceptualization; writing – review and editing. **Anne Hoppe:** Conceptualization; writing – review and editing. **Karine Levieux:** Conceptualization; writing – review and editing. **Martine Morisset:** Conceptualization; writing – review and editing. **Frédéric Valla:** Conceptualization; writing – review and editing. **Sibylle Blanc:** Conceptualization; writing – review and editing; methodology; supervision; project administration; validation. **Anaïs Lemoine:** Conceptualization; writing – original draft; writing – review and editing; methodology; data curation; visualization; supervision; project administration; formal analysis; investigation; validation.

## FUNDING INFORMATION

The authors have nothing to report.

## CONFLICT OF INTEREST STATEMENT

The authors declare no conflict of interest.

## Supporting information


Appendix S1.

## Data Availability

Data sharing not applicable to this article as no datasets were generated or analysed during the current study.
