# Supplementary material for: Oral food challenges in FPIES: A narrative review and proposals for emergency and home‐based management of acute FPIES reaction
Source: Pediatr Allergy Immunol. 2026 Jul 19;37(7):e70402. doi: 10.1111/pai.70402 (PMC13381817; doi:10.1111/pai.70402)
Supplement: Supplementary file 1 — Appendix S1. [file PAI-37-e70402-s001.docx]

# Appendix 1: Information sheet for parents and children during the FPIES-specific food reintroduction test

**Information sheet for parents and children during the FPIES-specific food reintroduction test**

Dear parents,

Your child will be taken for an oral food challenge. This document is intended to inform you about the progress of the test, in order to be able to obtain your consent. We ask you to read it carefully. The doctors and nurses are at your disposal for any further clarification.

What is the FPIES-specific oral food challenge?

Food protein-induced enterocolitis syndrome (FPIES) is a particular form of food allergy typically manifesting, in its acute form, by repeated vomiting occurring 1 to 4 hours after ingestion of the suspect food, sometimes accompanied by asthenia and pallor, followed by diarrhea appearing within 24 hours, and regressing after 24 hours of exclusion of food.

The oral food challenge (OFC) consists of eating a certain quantities of a food to which your child is supposed to be allergic to confirm the food allergy or seek to cure it.

A recent review of published studies on the modalities of OFC specific to FPIES (Bird JA, AAAI 2021) was able to conclude that a dose corresponding to 25-33% of an age-appropriate portion is sufficient to induce the onset of symptoms of acute FPIES in most cases, if there should be a reaction. This protocol reduces the risk of a severe reaction that could be induced by taking a full serving. As symptoms occur between 1-4 hours after ingestion of the food, monitoring for at least 4 hours after ingestion of the food should be expected.

Before performing the OFC, it is necessary to ensure the absence of IgE-mediated sensitization to the food using an immediate reading skin prick- test. If the latter is positive, a more gradual dose escalation protocol corresponding to the OFC protocol for IgE-mediated reactions is recommended, followed by monitoring for at least 4 hours.

Before the OFC is carried out, your child will be examined by a doctor. An intravenous line will be placed. During the test, your child will be under constant supervision. This monitoring will make it possible to respond as soon as a reaction appears and before the occurrence of more severe manifestations. The medical team is ready to intervene in the event of any symptoms suggestive of an allergy. In this case, adequate treatment will be administered. The occurrence of an allergic reaction may require prolonged hospitalization, sometimes in an intensive care unit.

Important:

It is important to stop all antihistamine treatment 7 days before hospitalization. Basic treatments for asthma should not be stopped. The test cannot be carried out in the event of uncontrolled asthma or allergic rhinitis or an infectious episode, particularly if it is digestive. If this is the case, please notify the service in advance so that the test can be postponed.

What should you do when you return home

Upon discharge, you will be given a prescription for an emergency reaction kit, including an oral rehydration solution and oral ondansetron. These medications should be obtained before continuing the reintroduction at home.

The treatment protocol to follow in the event of a reaction will be reviewed with you before discharge.

If there is no reaction during day hospitalization, you will be asked to give your child, at home and on another day, whether consecutive or not, a dose corresponding to 50% of the age-appropriate portion, then on a 3rd day, whether consecutive or not, a dose corresponding to a full age-appropriate serving. Administration at home should preferably be carried out in the morning or otherwise at midday, with parental supervision for at least 4 hours.

Either:

Day 1: 50% of an age-appropriate portion:

Day 2: 100% of an age-appropriate portion:

In the event of a reaction at home, it will be treated according to the following protocol:

- In the event of a mild reaction and no history of moderate or severe reaction, it is recommended to administer ondansetron orally or sublingually, if possible, followed by a trial of oral rehydration 20 minutes after vomiting as well as monitoring for signs of dehydration. If vomiting persists, the reaction then becomes moderate and/or severe depending on the general condition of your child and requires medical advice.
- In the event of a moderate reaction or a history of moderate reaction requiring intravenous rehydration in a hospital setting, it is recommended to administer ondansetron orally, if possible, and attempt oral rehydration 20 minutes later while waiting for emergency consultation.

• In the event of a severe reaction or a history of severe reaction with the need for prolonged hospitalization, it is recommended to administer ondansetron orally or sublingually, if possible, and to call the emergency medical service and attempt rehydration by oral route while waiting for medical care

This document must be signed and given to the nurses in the department before taking the test

I/we, the undersigned, legal representative(s),

***Parent 1*** *: (Name surname) ___________________________________________________________*

***Parent 2*** *:* *(Name Surname)* ___________________________________________________________

***Of the child***: *(Name Surname)* : _________________________________________________________

Born: __ __ / __ __ / __ __ __ __

Attest that I/we have:

- read the explanatory information sheet and agree to the oral reintroduction test on my/our child.
- been informed by Doctor ……………………………………………. of the terms of the oral reintroduction test, and the risks and benefits for my/our child during this test.
- asked all the questions that I/we deem useful and have clearly understood the answers.

Consequently, I/we give my/our consent to the practice of any diagnostic and therapeutic procedure required by the allergies presented by my/our child.

***Parent 1*** ***Parent 2***

**Date : 20__ __ / __ __ / __ __ Date : 20__ __ / __ __ / __ __**

**Signature : Signature :**
